# From **“**assistant” to **“**autonomous”: legal liability and ethical traceability frameworks for generative AI in clinical misdiagnosis scenarios, with a special focus on paediatrics

**DOI:** 10.3389/fdgth.2026.1849190

**Published:** 2026-06-30

**Authors:** Shiyi Xu

**Affiliations:** Capital Medical University Second Clinical School, Capital Medical University, Beijing, China

**Keywords:** artificial intelligence ethics, clinical decision support, generative AI, health policy, medical liability, paediatric patient safety, paediatrics

## Abstract

The integration of Large Language Models (LLMs) into clinical workflows has blurred the boundary between passive decision-support tools and systems capable of autonomous diagnostic reasoning. Current regulatory instruments—including the US FDA Clinical Decision Support Software (CDSS) guidance and the EU Artificial Intelligence Act (Regulation EU 2024/1689)—classify these systems primarily as “decision support,” yet the non-deterministic outputs and emergent reasoning of generative AI introduce liability complexities these frameworks were not designed to address. This hypothesis and theory article examines the regulatory gap surrounding LLM-associated clinical errors. Drawing on regulatory precedents, case law, and a representative scenario of polypharmacy mismanagement, we analyze the risks of automation bias and the structural inadequacy of the Learned Intermediary Doctrine when applied to generative AI. We advance the autonomy–liability correspondence hypothesis: legal liability should be allocated as a monotonic function of a system's structural autonomy from meaningful human review. From this we derive a “Three-Tiered Liability Escalation Framework” (T-LEF) that allocates accountability among AI developers, healthcare institutions, and clinicians according to measurable degrees of autonomy. Arguing that traditional Explainable AI is technically insufficient for foundation models, we propose Clinical Algorithmic Audit Trails (CAAT)—a cryptographically secured, privacy-preserving traceability infrastructure—as a complement to explainability, uncertainty estimation, human-factors evaluation, and post-market surveillance, alongside a structured Safe Harbor provision to support compliant innovation. Because paediatric patients—from neonates to adolescents—constitute a population in which these failure modes are both more probable and more consequential, we give paediatric care explicit focus: under-representation of paediatric data in training corpora, weight- and development-based dosing across narrow therapeutic windows, and children's inability to detect or contest erroneous recommendations jointly intensify these concerns, and a dedicated section examines how the T-LEF, the Clinically Deceptive Hallucination (CDH) evidentiary test, and CAAT apply to paediatric deployment. We acknowledge that the T-LEF and CAAT remain prospective proposals requiring empirical validation, stakeholder consultation, and refinement before adoption. This framework is offered as a structured contribution to regulatory deliberation, not a finalized implementation blueprint.

## 1 Introduction

The role of artificial intelligence in clinical practice has evolved considerably over the past decade. Early deployments were largely confined to narrow, task-specific algorithms—image-based pathology classifiers, rule-based drug interaction checkers, or structured alert systems—where the relationship between algorithmic output and clinical action was relatively direct and auditable. The emergence of generative Large Language Models (LLMs), including Med-PaLM 2 and GPT-4, has introduced a qualitatively different dynamic: these systems generate extended diagnostic narratives, synthesize complex patient histories, and produce recommendation outputs that can influence clinical judgment in ways that earlier tools could not (1, 2).

This shift from narrow assistance to generative reasoning creates a specific accountability problem. Studies on automation bias—the tendency of human operators to over-rely on automated recommendations, particularly under time pressure—have documented this phenomenon across aviation, nuclear operations, and clinical settings (3). When an LLM generates a plausible but factually incorrect clinical recommendation, and a clinician acts on it without independent verification, the existing mechanisms for assigning malpractice or product liability face structural difficulties (4, 5). The FDA's Manufacturer and User Facility Device Experience (MAUDE) database has recorded a growing number of adverse event reports implicating AI-assisted clinical decision support tools, indicating that LLM-associated clinical errors are no longer purely hypothetical (6).

The central problem this article addresses is the mismatch between the assumptions embedded in current medico-legal frameworks and the operational characteristics of generative AI. Existing frameworks were designed for deterministic tools with traceable outputs; they are poorly suited to systems whose outputs are non-deterministic, whose reasoning processes are opaque, and whose linguistic fluency may structurally impede independent clinician verification. We further emphasize that the proposed framework operates in the liability layer, complementing rather than substituting for the regulatory layer. This article does not claim to resolve that mismatch definitively. Rather, it proposes a structured framework—the T-LEF—as a basis for regulatory deliberation, and identifies the technical and governance infrastructure—CAAT—that any enforceable accountability system would require.

### 1.1 Central theoretical claim and specific contributions

The central theoretical claim of this article is that legal liability for harm arising from clinical generative AI should be allocated as a monotonic function of the system's structural autonomy from meaningful human review, rather than as a function of the system's nominal regulatory classification or its sophistication of output. We refer to this as the autonomy–liability correspondence hypothesis. Four subsidiary propositions follow from this central claim. First (T-LEF, Section 4), the gradient of autonomy can be operationalized into three discrete tiers that map onto distinct primary-liability parties. Second (CDH, Section 4.4), at the intermediate tier, partial liability shift from clinician to developer should be triggered by a structured evidentiary test that distinguishes algorithmic deception from ordinary clinical error. Third (CAAT, Section 6), the technical precondition for enforcing such graduated liability is not algorithmic explainability but cryptographically secured traceability of model provenance, retrieval grounding, prompt–output records, and clinician interaction metadata; CAAT is positioned as a complement to, not a replacement for, existing explainability and human-factors approaches. Fourth (Safe Harbor, Section 5), strict liability at the highest autonomy tier should be paired with a structured Safe Harbor at the intermediate tier, conditioned on transparent disclosure, architectural safeguards, audit infrastructure, and regulatory conformity, in order to preserve incentives for compliant innovation. The remainder of the article develops each of these propositions in turn and concludes with an explicit validation agenda (Section 9.1).

### 1.2 A note on paediatric salience

The analysis in this article applies to clinical generative AI across patient populations, but its motivating concerns are most acute in paediatric care, spanning the neonatal, infant, child, and adolescent periods. Three features make paediatrics a focal case. First, paediatric clinical data are systematically under-represented in the corpora on which foundation models are trained, and empirical evaluation of a general-purpose large language model against paediatric case challenges reproduced the correct diagnosis in only a small minority of cases, with an error rate of roughly 83 percent—substantially worse than reported adult-case performance. Second, paediatric therapeutics depend on weight-, age-, and maturation-based dosing across narrow and rapidly changing therapeutic windows, and a large share of paediatric prescribing is off-label, with dosages extrapolated from adult literature; medication errors in this population, though occurring at rates broadly comparable to adults, carry a substantially greater potential to cause harm. Third, infants and young children cannot themselves recognise, question, or refuse an erroneous recommendation, so the verification asymmetry that current liability doctrine presumes is displaced even further from the patient. For these reasons we treat paediatrics as a focal application throughout the article and develop its specific implications in a dedicated section (Section 8).

## 2 The regulatory landscape: existing frameworks and their limitations

### 2.1 FDA CDSS guidance and the “independent review of the basis” problem

The FDA's Clinical Decision Support Software guidance, updated in 2026, establishes a regulatory pathway for certain non-device CDS functions where, among other requirements, the healthcare professional can independently review the basis for the recommendation rather than rely primarily on the software output (7). This exemption rests on an assumption of transparency: that the clinician has meaningful access to the reasoning underlying the recommendation and can evaluate it against their own clinical judgment.

Taken seriously, the “independently review the basis” requirement is failed by generative LLMs in three specific ways. First, for LLM-generated clinical reasoning, the “basis” is the model's internal token-by-token probability distribution, which is not surfacable as an inspectable reasoning chain in any clinically meaningful sense. *post-hoc* rationalizations produced by the same model are not the model's actual reasoning; they are themselves model outputs subject to the same fluency-versus-accuracy asymmetry. Second, for RAG-generated citations, the apparent basis may be either fabricated outright or, more insidiously, real but used to support a claim the source does not actually support—a failure mode that is invisible to a clinician without independent verification of every cited primary source. Third, for probabilistic outputs, the user-facing presentation typically obscures the underlying uncertainty distribution, presenting a confident single recommendation that fails the spirit of the “independent review” requirement even when it formally complies with the letter of the guidance.

The FDA's proposed framework for AI/ML-based Software as a Medical Device (SaMD) acknowledges the challenge of “continuously learning” systems but does not provide specific provisions for the output variability characteristic of generative models (8).

### 2.2 The EU AI Act: high-risk classification without granular liability mechanisms

The EU Artificial Intelligence Act (Regulation EU 2024/1689), which entered into force in August 2024, classifies AI systems intended for medical use as “high-risk” and mandates conformity assessments prior to deployment (9). The Act operates through five distinct regulatory mechanisms that should not be conflated: (i) risk classification under Annex III; (ii) provider obligations including conformity assessment, quality management, and technical documentation under Articles 8–17; (iii) post-market monitoring obligations under Article 72, requiring providers to collect and analyze deployment-performance data; (iv) transparency duties under Articles 13 and 50; and (v) civil liability.

The AI Act is substantial on mechanisms (i)–(iv) but largely silent on mechanism (v). Civil liability has been left primarily to other instruments, especially the recast EU Product Liability Directive, Directive (EU) 2024/2853, which modernizes the EU strict-liability regime by expressly including software, including AI systems, within the scope of products covered by the Directive (10). The proposed AI Liability Directive was listed for withdrawal in the European Commission's 2025 Work Programme, reflecting the Commission's assessment that foreseeable political agreement was unlikely (11). This development increases the practical importance of the recast Product Liability Directive and national tort-law regimes in the European AI civil-liability landscape.

We emphasize that compliance with the AI Act's high-risk requirements does not, by itself, determine civil liability allocation in individual adverse events. These are related but doctrinally distinct mechanisms: regulatory compliance and liability allocation operate through different legal pathways, against different defendant parties, with different evidentiary standards, and with different remedies. The remainder of this article focuses on liability allocation, which the existing regulatory layer leaves substantially underspecified for generative LLM systems (12).

### 2.3 The learned intermediary doctrine: structural misapplication

A central structural problem in the current medico-legal framework is the application of the Learned Intermediary Doctrine (LID) to AI-assisted clinical decisions. The LID, developed in US pharmaceutical product liability law, holds that a manufacturer's duty to warn runs to the prescribing physician rather than directly to the patient, on the assumption that the physician serves as a knowledgeable intermediary capable of evaluating product risks (13).

The LID rests on two distinct rationales. The first is informational: the prescribing physician is presumed to possess the clinical training necessary to translate population-level product warnings into individualized risk–benefit assessment for the patient before them. The second is practical: in the era for which the doctrine was developed, direct manufacturer-to-patient warnings were neither feasible nor likely to be useful. Both rationales fit imperfectly when transposed to generative clinical AI (14). The informational rationale presumes a verification asymmetry favoring the clinician; in the case of an LLM that produces a fluent but fabricated evidentiary chain, the asymmetry runs in the opposite direction, with the clinician structurally less able than the developer to detect the failure mode under realistic workflow conditions. The practical rationale is simply not engaged: the AI system is deployed into the clinician's workspace by the same channel as any other software product. We therefore characterize the contemporary application of LID to opaque generative AI not as an error of application within the doctrine, but as a categorical mismatch between the doctrine's constitutive assumptions and the operational reality of the technology.

Peer-reviewed legal scholarship reaches consistent conclusions. Sullivan and Schweikart (15) document the inadequacy of existing tort doctrines for addressing injury caused by opaque AI systems, arguing that the LID effectively shields developers from liability for failure modes that the clinician has no practical means of detecting. Maliha and colleagues (16) analyze the broader question of liability redistribution across the AI ecosystem and conclude that traditional malpractice-centered regimes are inadequate to encourage both safe implementation and disruptive innovation. Recent doctrinal developments illustrate the unsettled state of the underlying law: in Himes v. Somatics, LLC, 549 P.3d 916 (Cal. 2024) (17), the California Supreme Court reaffirmed the Learned Intermediary Doctrine while expanding the plaintiff's pathways to prove causation in failure-to-warn cases, holding that a plaintiff need not show that a stronger warning would have altered the physician's prescribing decision and may instead show that the physician would have communicated the warning and that an objectively prudent patient would have declined treatment. While Himes concerned a medical device rather than an AI system, it illustrates that the doctrine's contemporary application is being actively reshaped by courts in directions that increase the salience of patient-centered causation analysis—an analysis that maps awkwardly onto AI-assisted decisions in which neither manufacturer-to-patient nor physician-to-patient communication channels are well-defined.

Three points warrant explicit acknowledgement. First, the LID is a US tort-law construct with no exact analogue in EU Member State civil-law systems, which typically operate through a combination of professional negligence and the EU Product Liability Directive. Second, the application of strict product liability to software has been historically unsettled, with the recast EU Product Liability Directive (Directive EU 2024/2853) now explicitly including software and AI systems within the definition of “product,” while many US states continue to treat software ambiguously and Commonwealth jurisdictions have generally declined to extend strict product liability to software absent statutory intervention. Third, hospital-integrated systems raise the further question of whether the deploying institution's modification of the system (custom prompts, RAG corpora, local fine-tuning) breaks the chain of “product” liability altogether and shifts the analysis toward enterprise or institutional liability. The T-LEF framework proposed in this article is designed to be doctrinally portable across these variations, but its precise implementation will require jurisdiction-specific calibration.

### 2.4 Jurisdictional fragmentation

The regulatory gap is compounded by the absence of international harmonization. The EU AI Act, the FDA's SaMD framework, and national regulations in jurisdictions including the United Kingdom, Canada, and China operate under different risk classification systems, conformity assessment requirements, and liability standards. This fragmentation creates conditions for regulatory arbitrage, particularly in the deployment of AI diagnostic tools in low- and middle-income countries (LMICs) with limited regulatory infrastructure—a concern addressed in Section 7.

## 3 A motivating clinical scenario: polypharmacy mismanagement

To illustrate the inadequacy of current frameworks, we present an illustrative clinical scenario drawn from documented patterns of AI-associated clinical errors in the literature (4, 5, 18). This scenario is explicitly an illustrative composite; it does not describe any specific patient or institution, and it is offered as a structured illustration of a failure mode rather than as a report of a documented adverse event.

An 82-year-old patient with newly diagnosed non-valvular atrial fibrillation, body weight of 58 kg, and serum creatinine of 1.7 mg/dL is evaluated in a high-volume outpatient cardiology clinic. The attending physician consults a hospital-integrated generative LLM to synthesize the patient's Electronic Health Record (EHR) and recommend an anticoagulant dosage regimen. The LLM accurately extracts the patient's age, weight, renal function, and atrial fibrillation diagnosis, but generates a hallucinated guideline recommendation suggesting apixaban 5 mg twice daily. The output includes a plausible-appearing but fabricated rationale asserting that dose-reduction criteria are not met and cites non-existent clinical guideline language to support the standard-dose recommendation.

Operating under time pressure in a high-volume clinical workflow, the physician reviews the seemingly well-supported rationale and approves the dosage. The patient subsequently suffers a severe hemorrhagic event requiring intensive care admission.

### 3.1 Clinical basis for the inappropriateness claim

This medication-safety example is designed to remove clinical ambiguity. For stroke prevention in non-valvular atrial fibrillation, the prescribing information for apixaban recommends dose reduction to 2.5 mg twice daily when a patient meets at least two of the following three criteria: age ≥ 80 years, body weight ≤ 60 kg, or serum creatinine ≥ 1.5 mg/dL (19). The patient in this scenario satisfies all three criteria. Accordingly, the AI-generated recommendation of apixaban 5 mg twice daily is not presented as a borderline dosing judgment, but as a clear medication-safety error amplified by a fabricated evidentiary rationale.

Under current legal doctrine, the physician bears primary malpractice liability for failing to independently verify the AI's fabricated citation against primary literature. This framing is analytically incomplete. It does not account for the mechanism by which the error occurred: the LLM's linguistic fluency enabled it to generate a structurally credible evidentiary chain that was factually false. This failure mode—which we term a “Clinically Deceptive Hallucination” (CDH)—is operationally distinct from a simple factual error and, we argue, warrants a distinct legal response.

To test whether T-LEF generalizes beyond the polypharmacy case, we offer three short companion vignettes.

### 3.2 Scenario A — sepsis early-warning system (tier 2 with CDH)

A hospital-deployed sepsis early-warning LLM produces an alert recommending broad-spectrum antibiotic initiation for a post-surgical patient, citing a “MEWS-style trajectory” and quoting a specific laboratory trend (“rising lactate from 2.1 to 3.8 over six hours”). The clinician initiates antibiotics; the cited lactate trend is later discovered to have been fabricated by the model, with no corresponding values in the EHR. The patient develops Clostridioides difficile colitis. Under T-LEF, this is a Tier 2 system (clinician approval required) with a strong CDH signal: factual incorrectness with material harm potential (criterion i), structural deception via fabricated laboratory citation (criterion ii), and a plausible verification-infeasibility claim given alert-fatigue condition (criterion iii). Proportional shared liability with developer contribution is the indicated outcome.

### 3.3 Scenario B — mental health chatbot in primary care triage (tier 2 borderline tier 3)

A primary care clinic deploys an LLM-based triage chatbot that produces structured suicide-risk assessments based on patient-reported symptoms. The chatbot returns a “low-risk” classification for a patient who subsequently dies by suicide, with the chatbot's rationale citing a non-existent validated screening tool. Although the chatbot output is nominally subject to clinician review, the workflow assigns review only when the output exceeds a defined risk threshold, and the empirical clinician acceptance rate for “low-risk” outputs is 99 percent over the preceding twelve months. Under T-LEF, this is a borderline case in which the system, although formally Tier 2, functions as Tier 3 in practice; the multi-factor classification analysis in Section 4.2 indicates mandatory reclassification review and may trigger the proposed Tier 3 patient-facing strict-liability pathway, subject to the downstream apportionment analysis in Section 4.5.

### 3.4 Scenario C — paediatric weight-based dosing (tier 2 with CDH)

A hospital-integrated generative LLM is consulted to recommend a gentamicin regimen for a 6-month-old infant weighing 7 kg with a febrile urinary tract infection and early renal impairment. The model correctly extracts the infant's age and weight but recommends an adult-pattern fixed dose, generating a plausible-appearing but fabricated rationale that cites a non-existent paediatric dosing table to justify it. The recommended dose is several-fold higher than the weight-appropriate regimen. Operating under time pressure, the clinician approves the order, and the infant sustains acute ototoxic and nephrotoxic injury. Because paediatric data are under-represented in training corpora and weight-based dosing errors are both more difficult to recognize as implausible and more harmful in infants, this is a Tier 2 system exhibiting a strong CDH signal across all three criteria of Table 2: factual incorrectness with material harm potential (criterion i), structural deception via a fabricated paediatric dosing citation (criterion ii), and a plausible verification-infeasibility claim where point-of-care paediatric dosing references are not uniformly accessible (criterion iii). Proportional shared liability with developer contribution is the indicated outcome, and the case is analyzed further in Section 8.

These three vignettes ground the framework in concrete contexts beyond polypharmacy and illustrate the interaction between T-LEF tier classification, the CDH evidentiary test, and the Safe Harbor and joint-causation analyses.

## 4 The three-tiered liability escalation framework (T-LEF)

### 4.1 Design principles

The T-LEF is proposed as a prospective compliance architecture rather than a punitive instrument. Its core design principle is the autonomy–liability correspondence hypothesis: liability allocation should track the degree to which a clinical AI system's autonomy structurally limits the clinician's capacity for independent verification and intervention. Three objective dimensions are proposed for tier classification: (1) the function of AI output in relation to clinical action (informational, recommendatory, or executory); (2) the nature of mandatory human review requirements; and (3) the applicable regulatory compliance standard.

A critical refinement, prompted by the reviewer's observation, is the distinction between formal and meaningful human-in-the-loop oversight. Formal oversight is satisfied by the presence of a technical review step in the workflow. Meaningful oversight additionally requires (a) sufficient time and contextual information to permit genuine evaluation of the AI output, (b) reviewer competence to evaluate it, and (c) absence of systematic over-acceptance such that the review step functions as a rubber stamp. The T-LEF tiers are defined with reference to meaningful oversight, not merely formal oversight, and the multi-factor classification analysis in Section 4.2 specifies how this distinction is operationalized.

### 4.2 Framework overview

**Figure 1:**
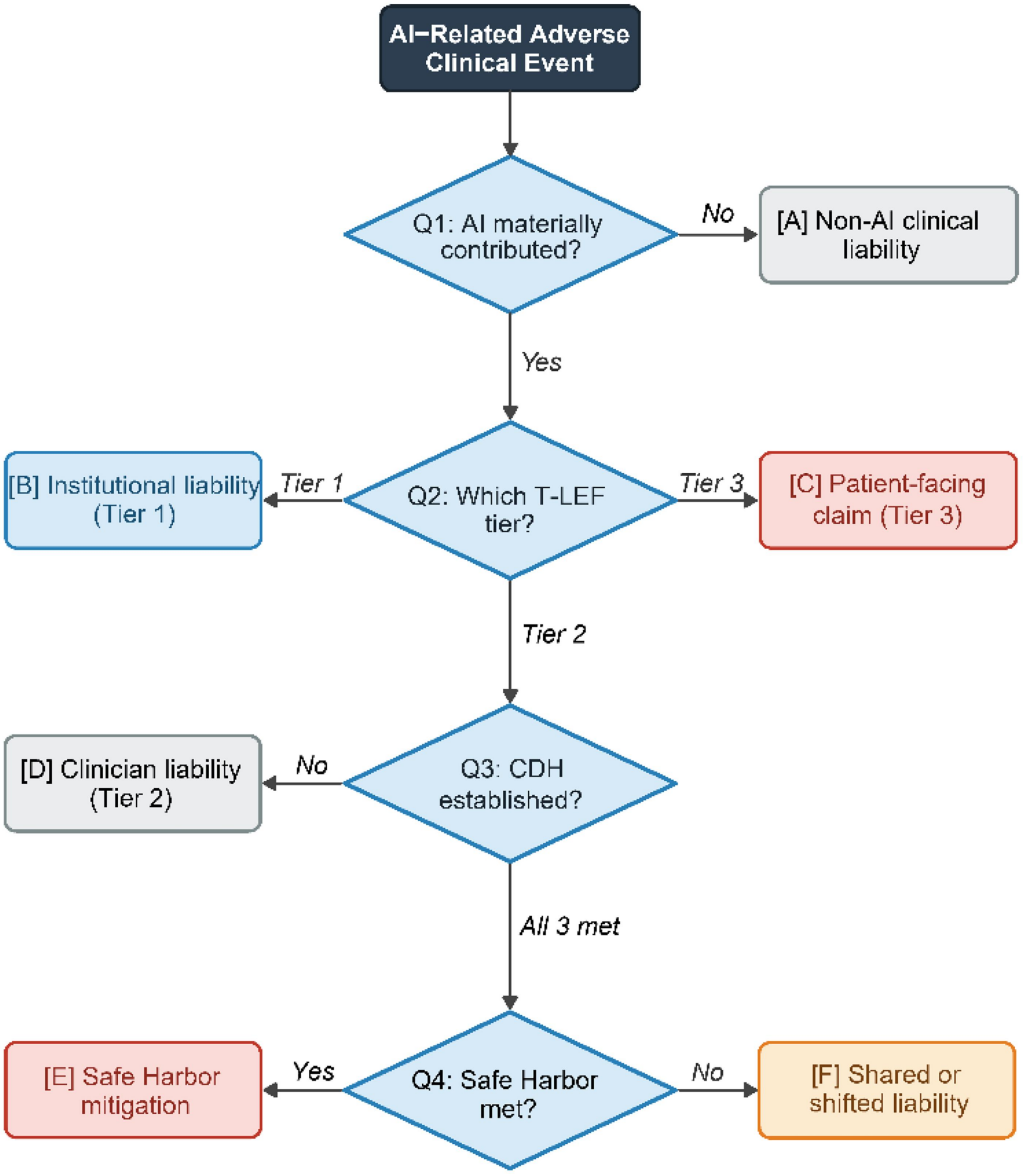
Sequential liability attribution pathway for AI-related adverse clinical events. The figure presents a simplified four-step decision sequence for applying T-LEF: AI contribution to harm, tier classification, CDH assessment, and Safe Harbor assessment. Abbreviated outcome boxes are used to improve readability, with explanatory detail provided in the caption rather than inside the flowchart. Outcome C refers to a proposed default patient-facing strict-liability claim in Tier 3 cases, while preserving downstream apportionment among developers, healthcare institutions, system integrators, EHR vendors, and local deployment teams where supported by CAAT records. In Tier 2 cases where CDH is established, satisfaction of Safe Harbor conditions leads to Safe Harbor mitigation, whereas failure to satisfy Safe Harbor conditions leads to proportional shared or shifted liability. CDH, clinically deceptive hallucination; T-LEF, three-tiered liability escalation framework; CAAT, clinical algorithmic audit trail.

Figure 1 presents a simplified decision pathway for applying T-LEF after an AI-related adverse clinical event. To improve readability, the figure separates the visual decision tree from the explanatory outcome legend. The figure is not intended to determine liability mechanically; rather, it structures the evidentiary sequence by asking whether AI materially contributed to harm, which autonomy tier applies, whether CDH is established in Tier 2 cases, and whether satisfaction of Safe Harbor conditions should mitigate developer-side liability.

The full operationalized classification criteria underlying the Q2 tier assignment node are presented in Table 1, which specifies the precise threshold criteria for each operational dimension across Tiers 1–3, providing the evidentiary basis for regulatory and judicial tier assignment decisions. As shown in Table 1, tier assignment is determined by the intersection of AI output function, the degree to which direct clinical action is triggered, and the presence or absence of mandatory human review—criteria that are, in principle, objectively assessable from system design documentation and CAAT records.

**Table 1:** Operationalized classification criteria for the three-tiered liability escalation framework (T-LEF).

Dimension	Tier 1: Administrative & Triage Autonomy	Tier 2: Augmented Clinical Decision Support	Tier 3: Autonomous Diagnostic & Therapeutic Intervention
AI Output Function	Information synthesis; routine communication drafting	Differential diagnosis generation; treatment recommendation	Direct triggering of clinical orders (e.g., medication dispensing, procedure initiation)
Direct Clinical Action	None; requires full human reformulation	Indirect; physician must actively approve	Direct; human-in-the-loop bypassed or functionally absent
Mandatory Human Review	Optional; best practice recommended	Mandatory; documented clinician sign-off required	Not applicable (no meaningful human oversight)
Primary Liability Party	Healthcare Institution	Shared (Clinician + Developer, proportionally)	Proposed default patient-facing claim against AI developer; downstream apportionment among developer, healthcare institution, system integrator, EHR vendor, and local deployment teams where supported by CAAT records.
Minimum Regulatory Compliance	ISO 27001; institutional QA protocols	FDA CDSS Tier B equivalence + CAAT mandatory	FDA PMA-equivalent; CE Class IIb/III; full CAAT
Safe Harbor Eligibility	Institutional accreditation	Published CDH rate + RAG architecture + CAAT integration	Not applicable; strict liability applies

The regulatory compliance standards listed in the “Minimum Regulatory Compliance” row are illustrative examples drawn from the FDA and EU regulatory contexts current at the time of writing. They are intended to indicate the type and stringency of compliance contemplated by each Tier rather than to specify mandatory thresholds; jurisdiction-specific implementation will be required.

Tier classification under T-LEF is the conjunction of five factors: (1) technical system design, (2) clinical workflow integration, (3) observed usage patterns, (4) institutional governance policy, and (5) applicable regulatory classification. No single factor is dispositive. The most consequential borderline case is the system that is formally Tier 2—the technical design includes a clinician-approval step—but operates as Tier 3 in practice because the approval step is informationally insufficient or because the clinician override rate is so low that the human-in-the-loop is functionally bypassed. We distinguish formal human-in-the-loop oversight from meaningful human oversight. Meaningful oversight requires (a) sufficient time and contextual information for genuine evaluation of the AI output, (b) reviewer competence to evaluate it, and (c) absence of systematic over-acceptance. Drawing on systematic-review evidence that clinician verification effort decreases monotonically as system acceptance rates rise (20), we propose, provisionally, that a Tier 2 system exhibiting clinician acceptance above 95 percent across a clinically significant denominator should trigger mandatory reclassification review under Tier 3. This threshold is a starting point for stakeholder discussion rather than a settled standard, and we propose its empirical calibration as part of the validation agenda in Section 9.1.

### 4.3 Tier 1: administrative and triage autonomy (institutional liability)

At this level, LLMs perform functions such as EHR summarization, appointment triage, or drafting routine clinical communications. Because the AI output does not directly recommend a specific clinical intervention and requires substantial human reformulation before any action is taken, clinical risk is relatively contained. Primary liability resides with the Healthcare Institution, which must be held accountable for implementing quality assurance protocols, managing training data governance, and preventing localized data drift that could systematically bias outputs for specific patient populations.

### 4.4 Tier 2: augmented clinical decision support (shared accountability)

This tier, detailed in the second column of Table 1, encompasses LLMs deployed for differential diagnosis generation, drug interaction analysis, or treatment protocol recommendation—functions that directly inform high-stakes clinical decisions. We propose a shared accountability model governed by the CDH construct.

A CDH is operationally defined as an AI-generated output satisfying all three criteria in Table 2. To distinguish CDH from adjacent failure modes:

**Ordinary hallucination:** factually incorrect output without structural deception (no fabricated citations or false confidence signaling). A competent clinician could reasonably detect ordinary hallucination under workflow conditions. CDH requires the additional structural and infeasibility criteria.
**Inaccurate recommendation:** a clinically defensible recommendation that turns out to be wrong in the individual case. CDH excludes good-faith clinical disagreement.
**Incomplete evidence synthesis:** an output that omits relevant evidence without fabricating false evidence. CDH specifically requires fabrication or false confidence signaling, not omission alone.
**Biased output:** an output reflecting systematic bias (e.g., demographic miscalibration). Bias and CDH are conceptually distinct and can co-occur, but neither implies the other.
**Non-reproducible LLM behavior:** output variability across identical or near-identical prompts. Non-reproducibility is a property of the system, not of any single output, and is addressed under Tier 3 considerations rather than under CDH.

This three-part test is intended to provide courts and regulators with a structured evidentiary standard that distinguishes CDH from ordinary clinical disagreement or straightforward factual error. We acknowledge that this test requires further validation: the threshold for “verification infeasibility” in particular will need to be calibrated through empirical study of clinician behavior under realistic workflow conditions, and the composition and methodology of the independent expert panel will require regulatory specification.

**Table 2:** CDH three-part evidentiary test for tier 2 liability apportionment.

Criterion	Description	Measurable Indicator
(i) Factual Incorrectness with Material Harm Potential	AI output contains clinically incorrect information capable of causing patient harm; established by independent expert panel	Severity ≥ NCC MERP Category D (“error reached patient and required monitoring or intervention to confirm no harm”) on harm-potential assessment; ≥ 2 of 3 expert panel members concur on factual incorrectness, following the majority-rule adjudication methodology standardized in clinical events classification practice (21).
(ii) Structural Deception	Output accompanied by fabricated citations, false confidence indicators, or plausible but unsupported reasoning chains	Presence of ≥ 1 of: (a) citation that cannot be matched to an existing source in CAAT RAG log or external citation databases; (b) explicit confidence assertion (“per guideline”, “standard of care”) without grounded source; (c) reasoning chain whose factual premises are unsupported by retrieved documents.
(iii) Verification Infeasibility	Output assessed as exceeding the reasonable verification capacity of a competent clinician under standard workflow conditions	Composite score across: (a) clinician time-per-decision under observed workload; (b) reading-level/token-complexity of the output relative to specialty norms; (c) accessibility of primary verification resources at the point of care (20). Preliminary threshold pending empirical calibration through clinician vignette experiments (see Section 9.1).

Under this framework: if a physician bypasses available verification mechanisms and acts on an LLM recommendation without documented critical appraisal, standard malpractice liability applies. If the LLM produces a CDH that satisfies the three-part test, developer-side liability may partially shift under the proposed T-LEF framework. The proportional allocation is determined by the degree to which each criterion is satisfied, providing courts with a graduated rather than binary instrument.

### 4.5 Tier 3: autonomous diagnostic and therapeutic intervention (proposed patient-facing strict-liability claim)

Tier 3, as defined in the third column of Table 1, involves AI systems that autonomously trigger clinical actions—such as initiating medication orders, adjusting device parameters, or generating actionable therapeutic instructions—without meaningful human review before clinical effect. In these scenarios, the clinically relevant feature is not merely the sophistication of the model, but the absence of a meaningful human checkpoint between AI output and patient-facing action (22).

We therefore propose strict product liability as a default patient-facing cause of action for Tier 3 adverse events. This proposal draws on established product-liability principles concerning defective products and on the increasing legal recognition that software may fall within product-liability regimes in appropriate statutory contexts (23). This proposal is not intended to establish the developer as the sole or final responsible party in every case. Rather, it is designed to provide injured patients with a clear and administrable route to compensation when harm results from autonomous AI action that occurs without meaningful human review. Downstream apportionment may still involve the foundation-model developer, deploying healthcare institution, system integrator, EHR vendor, local clinical informatics team, or other parties whose design, integration, configuration, or monitoring decisions materially contributed to the harmful output.

The rationale for this proposed default patient-facing claim is pragmatic and evidentiary. A negligence-only model would often require patients to prove breach using technical information they cannot realistically access, including training data, model configuration, evaluation records, retrieval architecture, or post-deployment monitoring data. A purely institutional-liability model would allocate primary responsibility to hospitals even when they lack the technical capacity to detect or prevent foundation-model failure modes. A pure no-fault compensation scheme could improve patient compensation but may weaken developer-side incentives for pre-deployment safety engineering. The proposed Tier 3 rule therefore operates as an initial patient-facing liability pathway, while preserving contribution, indemnification, comparative-fault analysis, or contractual risk allocation among the parties involved in system development and deployment.

The key distinguishing feature between Tier 2 and Tier 3, as highlighted in Table 1 under “Direct Clinical Action” and “Mandatory Human Review,” is not merely the sophistication of the AI output but the structural presence or absence of a meaningful human checkpoint before clinical action is taken. This distinction is operationally critical: a system that generates an autonomous medication order without physician approval is categorically different from one that generates a recommendation that a physician must actively endorse, regardless of the underlying model architecture.

CAAT records are central to this downstream apportionment analysis. They may show, for example, whether the harmful output resulted primarily from the foundation model, local fine-tuning, retrieval-corpus curation, prompt scaffolding, EHR-field mapping, disabled safety filters, or workflow changes that functionally removed human review. The Tier 3 proposal therefore separates the patient's initial route to compensation from the later allocation of responsibility among technically and institutionally distinct actors. This approach is consistent with general principles of comparative responsibility and apportionment, under which the patient-facing claim and the final distribution of responsibility among multiple causally contributing actors need not be identical (24).

## 5 The safe harbor provision: incentivizing compliant innovation

Critics of dynamic liability models argue that exposing AI developers to strict product liability will deter innovation, particularly for early-stage companies facing uncertain litigation risk. This concern merits engagement. However, the T-LEF does not propose unlimited liability; it proposes a structured Safe Harbor that creates a clear, achievable compliance pathway through which developers can substantially limit their Tier 2 liability exposure (see Table 1, Safe Harbor Eligibility row).

A developer shall receive a rebuttable presumption of non-defectiveness in any Tier 2 adverse event proceeding upon demonstrating, through documented evidence submitted to the relevant regulatory authority, satisfaction of all eight of the following pre-deployment standards:

- *Transparency Disclosure*: Public disclosure of model-specific CDH rates. During an interim phase, developers should report CDH rates against clearly described validation datasets. Once standardized CDH benchmark datasets are developed and publicly governed, Safe Harbor eligibility should require reporting against those standardized benchmarks, updated at minimum annually.
- *Architectural Safeguards*: Implementation of Retrieval-Augmented Generation (RAG) architecture with verifiable source grounding. We note explicitly that RAG does not eliminate false retrieval, unsupported synthesis, outdated sources, or incorrect reasoning over valid sources; architectural safeguards are necessary but not sufficient.
- *Audit Infrastructure*: Successful integration of a CAAT system meeting the minimum data standards specified in Section 6.
- *Regulatory Conformity*: Demonstrated compliance with applicable FDA CDSS Tier B guidance or EU AI Act high-risk conformity assessment requirements.
- *Independent Pre-deployment Validation*: Performance evaluation by an entity organizationally and financially independent of the developer, using a clearly described clinical validation set during the interim phase and, once available, a publicly governed standardized CDH benchmark.
- *Post-Market Monitoring*: Active surveillance of deployed model performance under Article 72 of the EU AI Act or analogous regimes, with predefined safety signal thresholds and response protocols.
- *Human-Factors Testing*: Evidence of pre-deployment evaluation under realistic clinical workflow conditions, including studies of alert fatigue, automation bias, and clinician override behavior.
- *Incident Reporting and Model-Update Documentation*: Mandatory reporting of clinically significant adverse events through MAUDE or equivalent national registries, and full version-controlled documentation of all model updates including the safety evaluation associated with each update.

The proposed Safe Harbor is modeled as an analogy rather than a direct doctrinal transplant. It draws from legal structures in which compliance with defined statutory or regulatory requirements can shape liability exposure, including the National Childhood Vaccine Injury Act and FDA medical-device quality-system regulation (25, 26). The critical distinction is that the T-LEF's Safe Harbor is conditioned on demonstrable transparency and verifiable risk mitigation, not on opacity. Shifting liability does not penalize innovation; it penalizes the externalization of algorithmic risk onto clinicians and patients without adequate disclosure or mitigation.

The benchmark component of this Safe Harbor proposal is prospective. Standardized CDH benchmark datasets do not yet exist as mature regulatory instruments. Their development would require coordinated work by regulatory authorities, clinical specialty societies, patient-safety organizations, clinical AI researchers, and patient representatives. Until such benchmarks are developed, CDH-rate reporting should be understood as provisional and should rely on transparent disclosure of dataset composition, case-selection criteria, expert-adjudication methods, and known limitations. Accordingly, the Safe Harbor should not be implemented as a complete legal defense until benchmark governance and validation procedures are established.

## 6 Clinical algorithmic audit trails (CAAT): technical architecture and implementation challenges

### 6.1 Traceability as necessary but not sufficient for accountability

Liability apportionment under the T-LEF requires robust, tamper-evident evidentiary mechanisms. Traditional Explainable AI (XAI) approaches—feature importance scores, attention weight visualization, SHAP value attribution—face well-documented limitations when applied to multi-billion parameter foundation models (27, 28). The computational cost of generating *post-hoc* explanations for each LLM inference in a high-volume clinical environment is prohibitive, and the resulting outputs are rarely interpretable by non-specialist clinicians or legal professionals.

We therefore propose that the policy framework treat algorithmic traceability as a necessary complement to algorithmic explainability, rather than as a replacement for it. Traceability answers the question “what happened?”—which inputs were submitted, which sources were retrieved, which output was produced, which clinician interaction occurred — and provides the evidentiary substrate that legal accountability requires. Explainability attempts to answer the question “why did it happen?” — which features or representations in the model caused the output — and remains important for safety improvement, even where it cannot serve as a primary basis for legal accountability.

CAAT, as developed in this article, is positioned as a complement to four governance layers:

**Explainability (XAI):** partial mechanistic understanding of model output, where technically achievable.
**Uncertainty quantification:** calibrated confidence and out-of-distribution detection.
**Human factors evaluation:** workflow integration, alert fatigue, and clinician interaction studies.
**Post-market surveillance:** longitudinal observation of model performance in deployment.

Each of these layers answers a distinct question; CAAT provides the evidentiary substrate that makes each legally actionable. Policy must therefore expand its demand from algorithmic explainability alone to a layered governance framework in which traceability sits alongside, not in place of, the other components.

### 6.2 CAAT minimum data standards

A CAAT system is defined as a continuously maintained, cryptographically secured, and privacy-preserving log of all clinically consequential LLM interactions. To be legally recognized under the T-LEF, a CAAT system must record, at minimum:

- *Model Provenance*: Cryptographic hash of the deployed model version and RAG knowledge base version at the time of inference.
- *RAG Citation Log*: Identification of specific source documents retrieved and used to ground the LLM's output, enabling *post-hoc* verification of whether cited sources exist and support the generated recommendation.
- *Prompt and Output Record*: Version-locked record of the input prompt (including EHR data fields queried, logged in tokenized or hashed form rather than as raw PHI where feasible) and the verbatim LLM output, stored with a tamper-evident timestamp.
- *Confidence Distribution Metadata*: The model's internal output probability distribution or calibrated uncertainty estimate, where technically accessible. Where the commercial model provider does not expose internal state, the developer must instead log calibrated *post-hoc* uncertainty estimates derived from external probes.
- *Clinician Interaction Metadata*: Documented record of the clinician's review action (approved, modified, or rejected), review duration, and any override rationale entered.

### 6.3 Technical implementation, governance, and feasibility

Blockchain-based immutable logging and Trusted Execution Environments (TEEs) have been proposed as mechanisms for ensuring CAAT data integrity. Research on blockchain-enabled EHR audit systems has demonstrated technical feasibility for access logging and tamper-evident record-keeping in healthcare contexts (29). Differential privacy techniques can be applied to audit log aggregation to prevent re-identification of individual patient data while preserving utility for adverse event investigation (30).

We address below the practical implementation questions raised in review.

***Log ownership:*** We propose a layered ownership model. The deploying healthcare institution holds primary custody of CAAT records, as the data controller under prevailing health-data law. The developer holds a derivative right to access records pertaining to its own model for safety surveillance, subject to data-minimization and contractual restrictions. Regulatory authorities and courts have conditional access through standard legal processes. This is analogous to the existing structure for EHR access and audit logs.
***Retention period:*** We propose alignment with prevailing medical-record retention requirements, which range from 7 to 30 years depending on jurisdiction and patient age at the time of care. CAAT records pertaining to pediatric patients should be retained at least until the patient reaches the age of majority plus the statute-of-limitations period in the relevant jurisdiction.
***Privacy protection:*** Three complementary mechanisms: (i) at-rest encryption of all CAAT records; (ii) differential privacy applied to aggregate audit queries, preserving utility for population-level safety surveillance while preventing re-identification; and (iii) tokenization or hashing of identifier fields, with the mapping table held separately under stricter access controls.
***Sensitive EHR data in prompts:*** Two safeguards: (i) institutional prompt-construction policies should restrict the EHR fields that may be inserted into LLM prompts to the minimum necessary for the clinical query; and (ii) CAAT logs should record the EHR field types accessed rather than verbatim content where feasible, with verbatim content reconstructable only under access conditions warranting it.
***Access during litigation, regulatory investigation, or quality review:*** We propose tiered access: (i) institutional quality review, accessible under existing peer-review protections; (ii) regulatory investigation, accessible under existing regulatory subpoena authority; (iii) litigation discovery, subject to existing rules of civil procedure and protective orders. CAAT does not create new access channels; it creates new content for existing access mechanisms.
***Closed commercial models:*** Where the commercial model provider does not expose internal model state, CAAT compliance is achieved through a combination of (i) developer-side logging of model version, fine-tuning state, and decoding parameters; (ii) deployer-side logging of all inputs and outputs at the API boundary; and (iii) externally derived uncertainty estimates from probe models. This is a second-best solution; full CAAT functionality requires developer cooperation, which the Safe Harbor structure is designed to incentivize.

Additional constraints must be acknowledged. Interoperability: current hospital information systems operate across heterogeneous EHR platforms with varying API standards, and CAAT integration would require standardized data exchange protocols that do not yet exist at scale. Computational and storage costs: continuous cryptographic logging of all LLM interactions in a high-volume clinical environment would impose significant infrastructure costs, particularly for resource-constrained health systems. Cross-jurisdictional legal standards: the legal admissibility of cryptographic audit records varies across jurisdictions and would need to meet different evidentiary standards in US, EU, and other legal contexts.

These challenges do not invalidate the CAAT proposal, but they indicate that implementation would require phased rollout, substantial regulatory coordination, and dedicated infrastructure investment. The analogy to financial transaction auditing and pharmaceutical clinical trial data integrity systems—where comparable logging requirements have been successfully implemented—suggests that these challenges are tractable, but the timeline and resource requirements should not be underestimated.

## 7 Global health equity implications

We explicitly disclaim the position that high-income-country liability standards can simply be exported to LMIC settings. Doing so without attention to local infrastructure, governance capacity, and cost burden risks reducing access to beneficial AI tools in precisely the settings where physician shortages make them most valuable. WHO estimates indicate physician shortages of up to 4.3 million health workers in LMICs, creating strong incentives for LLM-based primary care triage and diagnostic support adoption in contexts with limited regulatory infrastructure (31). AI diagnostic tools are actively being piloted in sub-Saharan Africa, South and Southeast Asia, and Latin America, often without the independent clinical verification capacity that high-income deployment contexts assume.

Without internationally harmonized liability standards, this deployment pattern creates conditions for regulatory arbitrage: developers may deploy high-risk diagnostic LLMs in regions with limited oversight, externalizing the clinical risk of hallucination-induced errors onto vulnerable patient populations. Analogous dynamics have been documented in the deployment of substandard pharmaceutical products and unvalidated medical devices in LMIC contexts (32).

At the same time, the framework must engage with five specific feasibility constraints in LMIC deployment: Infrastructure costs of CAAT deployment, which may be prohibitive without external funding mechanisms. Governance capacity, including regulatory expertise to evaluate Safe Harbor compliance claims. Data localization requirements that may complicate cross-border CAAT access and developer-side surveillance. The differential cost-benefit calculus when the alternative to imperfect AI triage is no diagnostic capacity at all. The risk that high compliance costs entrench incumbent developers and exclude local AI development.

We propose three complementary policy interventions, with explicit attention to the above constraints:

- WHO-Mediated T-LEF Harmonization: The WHO should convene a multi-stakeholder process to develop an internationally recognized T-LEF equivalence standard, enabling mutual recognition of liability compliance certifications across jurisdictions, paralleling the International Council for Harmonisation (ICH) framework for pharmaceutical regulatory harmonization. The eight Safe Harbor elements specified in Section 5 may be adapted to local infrastructure capacity through this process.
- CAAT Capacity Building: International development institutions should fund CAAT infrastructure development in LMICs as a component of digital health investment portfolios. Open-source CAAT reference implementations, developed under WHO auspices, would lower the technical barrier to adoption for resource-constrained health systems.
- Prospective Extraterritorial Accountability: As a normative proposal, regulators in high-income jurisdictions could consider whether developers subject to their jurisdiction should maintain minimum CAAT and CDH disclosure standards for substantially similar clinical AI systems deployed globally. Existing instruments such as GDPR Article 3 and the territorial-scope provisions of the EU AI Act show that extraterritorial regulatory design is possible in some digital contexts (33), but they do not establish a ready-made liability pathway for clinical AI harms occurring in LMIC settings. Any extraterritorial accountability mechanism for clinical AI would therefore require further legal development, international consultation, feasibility assessment, and safeguards against shifting compliance burdens onto under-resourced health systems. The proposal advanced here is limited to a prospective disclosure and traceability obligation designed to reduce regulatory arbitrage, not to impose high-income-country liability rules wholesale on LMIC health systems.

## 8 Paediatric-specific considerations

The framework developed above is articulated for clinical generative AI in general, but its motivating concerns are sharpest in paediatric care, which spans the neonatal, infant, child, and adolescent periods. We therefore set out here, in a dedicated section, why paediatric deployment intensifies each element of the autonomy–liability correspondence hypothesis and how the T-LEF, the CDH evidentiary test, and CAAT require paediatric-specific calibration. The aim is not to create a parallel framework for children, but to specify how the single framework should be applied when the patient is a child.

### 8.1 Why paediatric care is a heightened-risk context

Three features distinguish paediatrics. First, paediatric clinical data are systematically under-represented in the corpora on which foundation models are trained, and empirical evaluation has found that a general-purpose large language model reproduced the correct diagnosis in only a small minority of paediatric case challenges, with an error rate of roughly 83 percent—substantially worse than reported adult-case performance (34). The non-deterministic, fluency-driven failure modes described in Section 2.1 are therefore both more frequent and harder to anticipate in paediatric use. Second, paediatric therapeutics rely overwhelmingly on weight-, age-, and maturation-based dosing across narrow and rapidly changing pharmacokinetic windows, and a large share of paediatric prescribing is off-label, with dosages extrapolated from adult literature. Medication errors in paediatric inpatients occur at rates broadly comparable to adults but carry a substantially greater potential to cause harm (35); a hallucinated dose that would be obviously implausible in an adult may fall within a superficially plausible range for a child, defeating the clinician's heuristic safeguards. Third, infants and young children cannot themselves detect, question, or refuse an erroneous recommendation. The informational rationale of the Learned Intermediary Doctrine (Section 2.3) is doubly strained: not only does the verification asymmetry run against the clinician in the case of a fluent fabrication, but the residual patient-side check is mediated by parents or guardians who are not clinically trained, displacing meaningful protection still further from the patient.

### 8.2 Implications for T-LEF tier classification

The distinction between formal and meaningful human-in-the-loop oversight (Sections 4.1–4.2) is especially consequential in paediatrics. Because paediatric dosing errors are both easier for a fluent model to render plausible and more harmful, the threshold at which a formally Tier 2 system should trigger mandatory Tier 3 reclassification review should, we propose, be calibrated more conservatively for paediatric deployments than the provisional 95-percent acceptance benchmark proposed for the general case. The paediatric-care vignette (Scenario C, Section 3) is the paradigmatic instance: a clinician-approval step that is nominally present but operates, under workload, as a rubber stamp does not constitute meaningful oversight of a weight-based paediatric order.

### 8.3 Implications for the CDH evidentiary test

Each of the three CDH criteria of Table 2 acquires paediatric-specific content. The harm-potential assessment under criterion (i) should be referenced to weight-based and developmental dosing windows rather than to adult thresholds. Structural deception under criterion (ii) is particularly insidious where a model fabricates a paediatric-specific dosing rule or cites a non-existent paediatric guideline, precisely because clinicians cannot rely on adult-trained intuition to flag the value as wrong. Verification infeasibility under criterion (iii) must be assessed against the reality that point-of-care paediatric dosing references are not uniformly available and that paediatric subspecialty expertise is unevenly distributed, especially outside specialist centers. We propose that the clinician-vignette experiments in the validation agenda (Section 9.1) include paediatric strata so that the verification-infeasibility threshold is calibrated separately for paediatric practice.

### 8.4 Implications for CAAT

As already noted in our discussion of retention (Section 6), CAAT records pertaining to paediatric patients should be retained at least until the patient reaches the age of majority plus the applicable statute-of-limitations period—materially longer than for adult records. In addition, CAAT logging should explicitly capture the EHR fields that drive paediatric dosing, most critically current weight, gestational or postnatal age, and renal and hepatic maturation indices, because these are precisely the inputs whose misextraction or hallucinated substitution produces the most dangerous paediatric errors. Recording these fields (in tokenized or hashed form where feasible) is what allows a later audit to distinguish a model that misread the weight from one that invented a dosing rule, a distinction on which Tier 2 apportionment turns.

### 8.5 Implications for the safe harbor

The transparency-disclosure element (Section 5) should require paediatric-stratified CDH rates rather than aggregate figures, because adult-pooled performance can mask poor paediatric performance given the data asymmetries noted above. Correspondingly, independent pre-deployment validation and, once available, standardized CDH benchmark datasets should include adequately powered paediatric strata spanning the neonatal-to-adolescent range. As a matter of principle, a system validated only on adult cases should not qualify for Safe Harbor protection when deployed in paediatric care.

Taken together, these refinements show that the autonomy–liability correspondence hypothesis is not merely transferable to paediatrics but is most strongly motivated there: the population in which generative AI is least well-evidenced, in which dosing tolerances are narrowest, and in which the patient is least able to self-protect is precisely the population for which graduated, traceability-grounded liability allocation matters most.

## 9 A validation agenda and limitations

### 9.1 A validation agenda

We propose five empirical pathways, each linked to a specific component of the framework, and recommend that an international consortium of academic medical centers, legal scholars, and regulatory bodies be convened to coordinate this work over a five-year horizon.

- Clinician vignette experiments to calibrate the CDH “verification infeasibility” threshold. We propose a factorial design with verification time, output complexity, and resource access as primary factors, stratified by specialty and clinician experience level. The endpoint is the empirical proportion of test outputs that competent clinicians can verify under each combination of conditions.
- Retrospective audit studies of AI-assisted clinical decision-making in institutions with extant logging, to estimate the empirical base rate of CDH-classifiable events and to identify failure-mode clusters. This would also generate a preliminary CDH-benchmark corpus.
- Legal expert Delphi panels to assess the inter-rater reliability of T-LEF tier assignment and CDH evidentiary determinations across common-law and civil-law jurisdictions. This would identify cross-jurisdictional convergence and divergence in the application of the framework.
- Pilot CAAT implementations at sentinel hospital sites with diverse EHR infrastructure, to evaluate technical feasibility, governance burden, and clinician acceptance under real workflow conditions.
- Simulation studies of developer behavior under counterfactual liability regimes, to estimate the innovation-impact effect of the Safe Harbor provision and to inform calibration of its eight elements.

### 9.2 Limitations

This article presents a normative policy framework rather than an empirically validated system. Several limitations must be explicitly acknowledged:

- *Hypothetical Case Scenario*: The polypharmacy scenario in Section 3 is a structured illustration of a documented failure mode, not a report of a specific case. While the failure mode it depicts has been characterized in the literature (4, 5), the specific scenario has not been empirically verified. Prospective documentation of real-world CDH events will be essential for validating the framework's assumptions.
- *Absence of Stakeholder Validation*: The T-LEF and CAAT proposals have not been evaluated through structured consultation with clinicians, hospital administrators, legal professionals, AI developers, or patient advocates. The framework's practical feasibility—particularly the CDH three-part test and CAAT minimum data standards—requires iterative refinement through multi-stakeholder engagement before regulatory adoption.
- *CDH Operationalization*: The CDH construct lacks an empirically validated measurement instrument. The “verification infeasibility” criterion in particular requires calibration through behavioral research on clinician decision-making under realistic workflow conditions. Without such calibration, courts applying the CDH test would face significant evidentiary uncertainty.
- *CAAT Technical Readiness*: While blockchain-based audit logging and differential privacy techniques have been demonstrated in analogous contexts, their integration into clinical AI infrastructure at scale has not been prospectively evaluated. Implementation costs, interoperability requirements, and governance structures remain to be specified through regulatory and technical working groups.
- *Jurisdictional Scope***:** Despite the addition of Section 2.3 acknowledging jurisdictional variation, the framework remains primarily anchored in US and EU regulatory contexts. Its applicability to other legal systems requires jurisdiction-specific analysis.
- *Innovation Impact Uncertainty*: The claim that the Safe Harbor provision adequately offsets the innovation-deterrent effect of strict Tier 3 liability is plausible but unverified. Empirical study of developer behavior under analogous liability regimes would strengthen this argument.

## 10 Conclusions

The medico-legal frameworks currently governing clinical AI were designed for deterministic tools with traceable outputs. Generative LLMs differ from those tools in ways that matter for liability: their outputs are non-deterministic, their reasoning processes are opaque, and their linguistic fluency may structurally impede independent clinician verification. The Learned Intermediary Doctrine, as currently applied, concentrates liability on the clinician while shielding developers from responsibility for failure modes that clinicians have limited capacity to detect.

The T-LEF proposed in this article offers a structured approach to this problem grounded in the autonomy–liability correspondence hypothesis: legal liability should be allocated as a monotonic function of structural autonomy from meaningful human review. The framework's core contributions are: (1) an operationalized tier classification system (Figure 1; Table 1) with explicit multi-factor criteria and the formal/meaningful oversight distinction; (2) the CDH construct (Table 2), which distinguishes structurally deceptive hallucination from ordinary hallucination, inaccurate recommendation, incomplete evidence synthesis, biased output, and non-reproducible LLM behavior; (3) the CAAT infrastructure, positioned as complementary to existing explainability, uncertainty, human-factors, and post-market surveillance layers; and (4) the eight-element Safe Harbor that preserves innovation incentives while requiring transparent risk mitigation. Importantly, the framework operates in the civil-liability layer and complements—rather than substitutes for—the regulatory layers established by the FDA, the EU AI Act, and the recast EU Product Liability Directive. Throughout, we have given paediatric care a special focus (Section 8): the population in which generative AI is least well-evidenced, in which dosing tolerances are narrowest, and in which the patient is least able to self-protect is precisely the one for which graduated, traceability-grounded liability allocation is most urgently needed.

We recommend: (i) the FDA and EU Commission jointly convene a working group to develop CAAT minimum data standards within 24 months; (ii) professional medical associations update clinical AI governance guidelines to incorporate CDH documentation requirements; and (iii) AI developers voluntarily adopt Safe Harbor compliance standards in advance of mandatory regulatory implementation; and (iv) an international consortium initiate the five-pathway validation agenda. By transitioning from a static product-liability model to a dynamic shared-accountability paradigm grounded in technical traceability and proportional liability, this framework aims to support both patient safety and responsible innovation in clinical AI.

## Data Availability

The original contributions presented in the study are included in the article/Supplementary Material, further inquiries can be directed to the corresponding author.
